# Financial impact of medication reviews by clinical pharmacists to reduce in-hospital adverse drug events: a return-on-investment analysis

**DOI:** 10.1007/s11096-023-01683-w

**Published:** 2024-02-05

**Authors:** Mégane Jermini, Caroline Fonzo-Christe, Katherine Blondon, Christelle Milaire, Jérôme Stirnemann, Pascal Bonnabry, Bertrand Guignard

**Affiliations:** 1 Pharmacy, Geneva University Hospitals, Rue Gabrielle Perret Gentil 4, 1205 Geneva, Switzerland; 2 Institute of Pharmaceutical Sciences of Western Switzerland, School of Pharmaceutical Sciences, University of Geneva, Geneva, Switzerland; 3 Medical and Quality Directorate, Geneva University Hospitals, Geneva, Switzerland; 4 Finance Department, Geneva University Hospitals, Geneva, Switzerland; 5 Division of General Internal Medicine, Department of Medicine, Geneva University Hospitals, Geneva, Switzerland

**Keywords:** Adverse drug event, Clinical pharmacy, Cost analysis, Drug related problem, Economic evaluation, Internal medicine, Medication review

## Abstract

**Background:**

Adverse drug events contribute to rising health care costs. Clinical pharmacists can reduce their risks by identifying and solving drug-related problems (DRPs) through medication review.

**Aim:**

To develop an economic model to determine whether medication reviews performed by clinical pharmacists could lead to a reduction in health care costs associated with the prevention of potential adverse drug events.

**Method:**

Two pharmacists performed medication reviews during ward rounds in an internal medicine setting over one year. Avoided costs were estimated by monetizing five categories of DRPs (improper drug selection, drug interactions, untreated indications, inadequate dosages, and drug use without an indication). An expert panel assessed potential adverse drug events and their probabilities of occurrence for 20 randomly selected DRPs in each category. The costs of adverse drug events were extracted from internal hospital financial data. A partial economic study from a hospital perspective then estimated the annual costs avoided by resolving DRPs identified by 3 part-time clinical pharmacists (0.9 full-time equivalent) from 2019 to 2020. The return on investment (ROI) of medication review was calculated.

**Results:**

The estimated annual avoided costs associated with the potential adverse drug events induced by 676 DRPs detected was € 304,170. The cost of a 0.9 full-time equivalent clinical pharmacist was € 112,408. Extrapolated to 1 full-time equivalent, the annual net savings was € 213,069 or an ROI of 1–1.71. Sensitivity analyses showed that the economic model was robust.

**Conclusion:**

This economic model revealed the positive financial impact and favorable return on investment of a medication review intervention performed by clinical pharmacists. These findings should encourage the future deployment of a pharmacist-led adverse drug events prevention program.

**Supplementary Information:**

The online version contains supplementary material available at 10.1007/s11096-023-01683-w.

## Impact statements

- This study highlighted the importance of employing an economic model that assesses costs comprehensively using real hospital costs for more accurate results and revealing the return on investment (ROI) of clinical pharmacy activities.
- The calculation model created is robust, innovative, easy to use and applicable in other healthcare context.
- This project confirmed that medication reviews performed by a clinical pharmacist in internal medicine wards prevent adverse drug event-related expenses and is cost-effective.
- Pharmacists should focus their interventions on patients at a high risk of adverse drug events or on drugs that can cause very expensive adverse drug events to ensure even greater cost-effectiveness.

## Introduction

Adverse drug events (ADEs) resulting from medication errors (MEs) have a significant negative impact on patient safety. However, they also contribute to rising health care costs, which have reached an estimated USD 177 billion in the United States, € 370 million in Sweden, and more than € 1 billion in Germany [1–3]. ADEs cause avoidable health care consumption through unplanned consultations with general practitioners or emergency departments, hospitalizations, and prolonged hospital stays. One study stated that 4 emergency department visits per 1000 inhabitants were related to ADEs [4]. Hospitalization rates due to ADEs vary from 3.25 to 25% [5–8], and the prevalence of in-hospital ADEs ranges from 3.2 to 5.6% depending on the country [6].

Clinical pharmacists can reduce the risks of ADEs by preventing medication errors (MEs) through optimizing drug therapy and improving patient medication adherence, thus indirectly reducing unnecessary expenditures [7–14]. Clinical pharmacists can also save money by negotiating drug purchase prices with suppliers, ensuring proper inventory management, and promoting the safe, efficient, appropriate, and economical use of pharmaceuticals within hospitals.

Most research on the economic dimensions of clinical pharmacists’ activities has been conducted in the United States. Systematic reviews have shown the positive economic impacts of hospital and community pharmacists’ activities [15–19]. Studies have also assessed the cost savings association with using less expensive drugs, promoting generic or biosimilar drugs, substituting parenteral drugs for oral forms, optimizing treatment duration, and enhancing medication management [20–29]. Additionally, studies measured cost avoidance through pharmacist-led interventions, such as patient-tailored activities to improve adherence or optimize drug therapy on wards [9, 20–23, 30–42].

Pharmacoeconomic models, such as cost minimization, cost-effectiveness, cost-benefit, or cost-utility analyses, are not easy to grasp and apply for pharmacists. They are not always clearly described in published studies and there is often significant between-study variability in healthcare settings, methodologies, and results. An absence of standardized economic evaluation methods in this field and the lack of comprehensive economic studies complicates data interpretation, limits reproducibility, and constrains the applicability of their results to different healthcare systems. Overcoming the challenge of reducing healthcare costs and demonstrating a return on investment (ROI) remains a major obstacle to expanding the employment of clinical pharmacists in many countries.

### Aim

This study aimed to develop a simple, easily applicable economic model based on literature and adapted to Switzerland’s healthcare system to demonstrate that clinical pharmacists’ medication review (MR) activities could reduce hospital expenditures by preventing costly ADEs among inpatients.

### Ethics approval

It was not necessary to undergo an ethical compliance evaluation procedure for a pharmacoeconomic study.

## Method

### Pharmaceutical intervention

This study defined pharmacist-led intervention as identifying drug-related problems (DRPs) through inpatient medication reviews and providing treatment optimization recommendations during ward rounds. According to the Pharmaceutical Care Network Europe (PCNE) [43] and Hepler and Strand definition [44], a DRP is an event or circumstance involving drug therapy that actually or potentially interferes with desired health outcomes. We considered that unsolved DRPs may have led to acute ADEs during hospitalization. ADEs and related costs occurring after discharge were not included in the study. The ADE definition of Bates et al. was employed in this research [45].

### Design, setting, and study population

To develop the economic model, two clinical pharmacists collected data on avoided costs related to different categories of DRPs over a 12-month period (January–December 2019) in a 200-bed internal medicine division of a Swiss university hospital. Five categories were chosen because of their frequent occurrence in a previous study conducted in the same department: *improper drug selection*, *drug interactions*, *untreated indications*, *inadequate dosages*, and *drug use without an indication* [46]. Only DRPs for which treatment optimization recommendations were implemented by physicians were considered in the analysis.

### Economic model development

#### Study perspective

The partial economic study evaluated the avoided costs associated with ADE prevention from a health care provider’s perspective. In this study, the hospital, which is publicly funded, was considered a payer and provider.

#### Selection of DRPs and identification of potential ADEs

Among the 538 DRPs detected by the two pharmacists in one year, 20 in each of the five categories of DRPs, each associated with a different clinical condition, were randomly selected. An expert panel (one attending physician in internal medicine and one senior clinical pharmacist) assessed the potential acute ADEs that could have occurred with the highest probability without the intervention of the clinical pharmacist for each of these 100 DRPs. They evaluated these 100 DRPs individually using their clinical experience, knowledge, and the medical literature. They estimated the probability of an ADE occurring for each DRP according to the stratification probability scale described by Nesbit et al. [21]. This scale assigns a probability of 0, 0.01, 0.1, 0.4, and 0.6 to occurrences (corresponding to no probability, very low probability, low probability, medium probability, and high probability, respectively). The most likely related potential ADE was matched to the probability of occurrence of each clinical DRP by consensus. In the absence of consensus, an attending physician specializing in clinical pharmacology could be asked to make the final decision. Examples of DRPs are presented in electronic supplementary material 1.

#### Avoided costs of the potential ADEs related to DRPs

The hospital’s cost accounting team calculated the avoided costs by preventing potential ADEs using real data on the direct hospital costs that would have been incurred due to additional medical care and/or extended length of stay. They use the standardized REKOLE® method [47], which establishes how each hospital stay consumes direct health resources, called *work units* (e.g., drugs consumed, minutes in the operating room, minutes of care), and indirect resources leading to expenses, called *unit costs of load centres* (which are groupings of expenses necessary to implement an activity, such as infrastructure, sterilization, anaesthesia, imaging, or cleaning). This microcosting method makes it possible to estimate the cost of a hospital stay as well as the additional costs of an extended stay or readmission.

To obtain an accurate representative cost for each ADE identified by the expert panel, it was considered translatable into a disease and was associated with a corresponding International Classification of Diseases version 10 (ICD-10) [48, 49] classification code. The accounting team extracted data on every hospital stay for each code of interest during 2017, and they analyzed and adjusted their costs to obtain a realistic median cost for the hospital associated with managing those conditions. Costs were calculated in Swiss francs.

#### Avoided costs by DRP category

A median cost was applied to each DRP’s potential ADE and weighted by that ADE’s probability of occurrence, as assigned by the expert panel. These were used to calculate the avoided cost for each of the 100 DRPs. Table 1 illustrates the calculation process using the example of an identified ADE. Once the avoided costs were known for each of the 100 DRPs, a mean and median avoided cost by DRP category could be calculated. The economic model used median costs because of the cost data’s non-Gaussian distribution. Costs were expressed in Swiss francs and were converted into Euros (€) using 2017’s mean exchange rate (€ 1 = CHF 1.11156946).

**Table 1:** Example calculation process for the avoided costs related to a prevented adverse drug event

DRP	Recommended pharmaceutical intervention	DRP category	Potential ADE	Probability of ADE occurrence	ADE cost (€)	Avoided cost (€)
Patient treated with 15 mg rivaroxaban once daily in the context of atrial fibrillation and renal function at 53 ml/min/1.73 m^2^	Increase the dose of rivaroxaban to 20 mg once daily	Inadequate dosage	Ischemic stroke	0.01	11,056	110

*ADE* adverse drug event, *DRP* drug-related problem, *€* Euros

Multiply the probability of occurrence with the avoided cost of the pathology to obtain the ADE avoided cost for this DRP: 0.01 × 11,056 € = 110 €

### Return on investment analysis

#### Investment costs

Investment costs were the hospital’s spending on medication review activities, mainly annual expenditure on clinical pharmacists’ salaries in 2019–2020. The part-time activities of the three pharmacists corresponded to a 0.9 FTE position.

#### Avoided costs

Avoided costs were the estimated hospital costs saved based on all the DRPs collected that led to prescription modifications over a one-year period (April 2019–March 2020) by three part-time clinical pharmacists. The previously estimated median avoided costs were applied to each clinical intervention implemented, according to their DRP categories, and cumulated.

#### Return-on-investment calculation

The clinical pharmacists’ economic impact was assessed by measuring the ROI of their medication review activities. The hospital’s annual investment in pharmacists’ salaries was subtracted from the annual net savings generated by ADE prevention. This resulted in a net saving with which to calculate an ROI ratio to the annual investment.

#### Sensitivity analyses

Three sensitivity analyses were carried out to assess the robustness of the economic analysis, to identify the study’s limitations and to understand how to best interpret the economic evaluation. The first analysis decreased the cost of any ADEs by 2.5 times (60%), the second analysis decreased their probability of occurrence by half, and the third analysis weighted 1.5 times (33.33%) decrease in costs of any ADE and fixed the probability of occurrence to 0.05.

## Results

### Economic model development

#### Avoided costs of the potential ADEs related to DRPs

The expert panel’s evaluation of 100 DRPs identified 33 different potentially preventable ADEs. Several DRPs led to the same ADE. These ADEs and the institutional costs calculated for their management are presented in electronic supplementary material 2. Median ADE costs ranged from € 591 to € 17,384, demonstrating that some of the DRPs detected could prevent ADEs that proved very costly to the hospital. Serious ADEs affecting a patient’s quality of life or causing severe complications were the most expensive in terms of hospital management (e.g., femoral neck fracture, osteomyelitis, intracerebral haemorrhage). The suggested probabilities of occurrence varied from 0 to 0.4, with none being assigned a probability of 0.6. The expert panel assessed that 29 of the 100 clinical situations would not cause immediate ADEs and were assigned a probability of occurrence of 0; 8 cases were assigned a probability of 0.01; 55 were assigned a probability of 0.1; and 8 cases were assigned a probability of 0.4.

#### Avoided costs by DRP category

**Fig. 1:**
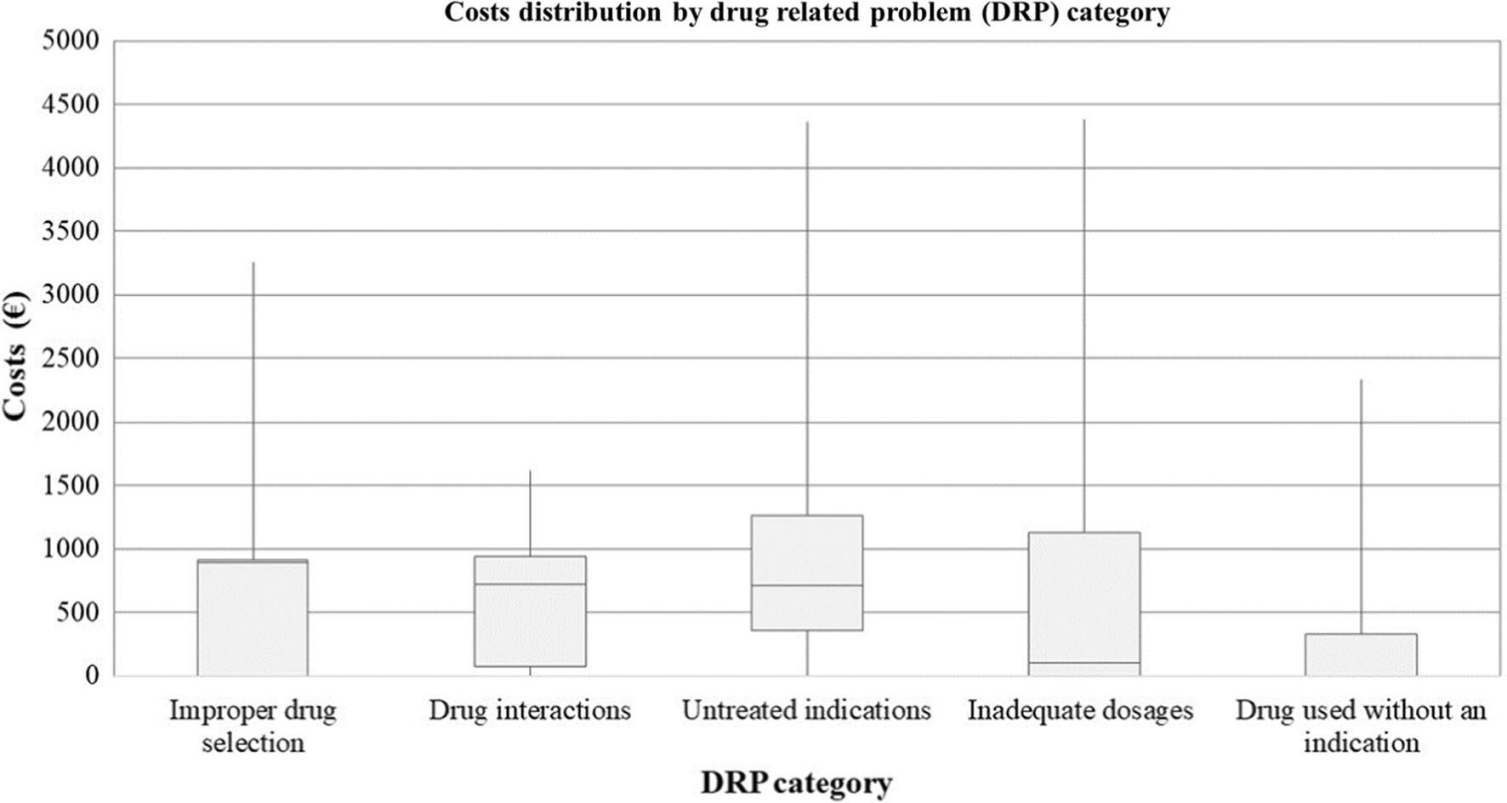
Boxplot distributions of different costs by category of drug-related problem, including median, inter-quartile range, upper and lower quartiles, and whiskers

Median costs associated with each category of DRP are presented in Table 2. They ranged from € 0 to € 897. The distribution of individual costs by DRP category is shown in Fig. 1.

**Table 2:** Mean and median avoided costs associated with each DRP category and their dispersion parameters

Dispersion parameters	Improper drug selection	Drug interactions	Untreated indications	Inadequate dosages	Drug use without an indication
*Costs (€)*					
Mean	695	611	910	772	313
Standard deviation	753	480	953	1259	600
Median	897	725	714	100	0
Minimum	0	0	0	0	0
Maximum	3251	1615	4354	4373	2331
1st decile (10%)	0	66	31	0	0
1st quartile (25%)	0	78	365	0	0
3rd quartile (75%)	932	942	1260	1135	328
9th decile (90%)	1093	1119	1423	1939	967

*€ Euros*

#### Return-on-investment analysis

Over 12 months, the three part-time pharmacists performed 144 medication reviews for 973 patients (6–7 patients per review), detecting and solving 676 DRPs. Of these, 184 involved *inadequate doses* (over- or underdosing), 178 involved *untreated indications*, 152 involved *drug interactions*, 108 involved *drug use without an indication*, and 54 involved *improper drug selection*. The total avoided cost of these 676 prevented DRPs, based on the median avoided costs by category, was estimated at € 304,170. Most of this was saved by identifying *untreated indications* (€ 127,146). Detecting DRPs involving *drug use without an indication* did not save the hospital money because most were associated with drugs that did not induce acute ADEs that might prolong hospitalization. The total avoided costs by DRP category are shown in Table 3*.*

**Table 3:** Annual number of DRPs detected by 0.9 FTE pharmacists, associated annual avoided cost by DRP category, and total avoided cost

DRP category	Number of DRPs	Annual cost saving by DRP category
Improper drug selection	54	€ 48,434
Drug interactions	152	€ 110,215
Untreated indications	178	€ 127,146
Inadequate dosages	184	€ 18,374
Drug use without an indication	108	€ 0
Total	676	€ 304,170

*DRP* drug-related problem, *€* Euros

The financial investment required to enable the three part-time clinical pharmacists (totalling a 0.9 FTE position) to carry out their medication review activities was € 112,408. The annual net savings were € 191,762. Extrapolating this result to 1 FTE clinical pharmacist’s position came to € 213,069, giving an ROI of 1–1.71.

#### Sensitivity analyses

The total annual avoided costs, net savings, and ROI were calculated using three sensitivity analyses, as presented in Table 4. The economic impact remained positive in the sensitivity analyses where costs were reduced by 60% and where the probability of occurrence was made very low. The third sensitivity analysis illustrated the economic model’s break-even point.

**Table 4:** Avoided costs, invested costs, net saving, and return on investment measured in sensitivity analyses

Economics parameters	Original ROI analysis	Sensitivity analysis 1 (Costs 2.5 × less)	Sensitivity analysis 2 (Probability divided by 2)	Sensitivity analysis 3 (Costs 1.5 × less and probability set at 0.05)
Avoided cost	€ 304,170	€ 121,621	€ 152,026	€ 112,452
Invested costs	€ 112,408	€ 112,408	€ 112,408	€ 112,408
Net saving (0.9 FTE)	€ 191,762	€ 9,213	€ 39,619	€ 44
Net saving (1.0 FTE)	€ 213,069	€ 10,237	€ 4,4021	€ 49
ROI	1.71 (171%)	0.08 (8%)	0.35 (35%)	0.004 (0.4%)
ROI (ratio)	1: 1.71	1: 0.08	1: 0.35	1: 0.004

*FTE* full-time equivalent, *DRP* drug-related problem, *ROI* return on investment, *€* Euros

## Discussion

This study demonstrated that medication reviews performed by clinical pharmacists during ward rounds could significantly reduce hospital costs by preventing ADEs. Our results showed a cost-saving ratio or ROI of €1.71 for each €1 invested in clinical pharmacy activities. Our calculated benefits fall within the range of findings from prior studies.

Few studies evaluated the ROI of clinical pharmacist activities in a hospital setting, and even fewer quantified the avoided costs associated with ADE prevention or calculated a median avoided cost for each DRP category. To the best of our knowledge, this study is the first to use real institutional costs from analytical accounting through a microcosting approach.

Major pharmacoeconomic reviews on clinical pharmacy interventions showed a wide range of ROIs from 1 to 75.84 [15–18], reflecting the variability in study types included, interventions evaluated and costs considered. For example, the research perspective adopted can impact the costs considered.

In Schumock and Perez’s review, the median ROIs ranged from 1 to 4.81 [16, 17], corroborating our own findings. Studies that monetized ADEs using costs per DRP category calculated an ROI ranging from 1 to 14 [8, 9, 21, 22, 50, 51]. All these studies used a slightly different DRP cost calculation method (e.g., fixed cost for any ADE; fixed ADE occurrence probability; costs saved directly when a drug was stopped or changed.).

Two European studies [22, 51] used the microcosting method developed by Rottenkobler et al. [3] to assign an avoided cost to a prevented ADE associated with a probability of occurrence score, as per Nesbit et al. [21]. The ROIs measured were 1–8.64 and 1–1.76. They fixed the value of a prevented ADE to be € 1,057 and € 1,079, respectively. Rottenkobler’s cost calculation method was quite similar to ours in that it incorporated the sum of the single cost components associated with inpatient treatments for ADEs based on cost centres. This way of quantifying ADEs enables more accurate and transparent cost assessments.

The differences between prior studies illustrated the variabilities in health care systems, settings, types of costs, cost calculations, or definitions of a pharmaceutical intervention or an ADE. This makes data comparisons, data extrapolations and generalizations challenging.

One strength of our study is the method of calculating avoided costs, which was closer to the reality on wards than what other researchers have used to date. We put values on ADEs using real costs derived from our hospital’s in-house accounting data rather than estimating costs from the literature. This was innovative because we calculated a median avoided cost for five DRP categories by costing real clinical situations at risk of ADEs and prolonged hospital stays. The actual hospital cost of managing each of these situations could be calculated by translating ADE into a disease or condition whose cost was easily quantifiable. The use of ICD-10 to translate an ADE is novel and practical as long as it is compatible with the health care cost valuation system of a country, as is the case in Switzerland.

Costs were saved in each category of DRP detected by the pharmacists, except *drug use without an indication*. Potential ADEs from this DRP category were calculated to have a median cost of € 0 because in more than 50% of the 20 cases quantified, the potential ADEs would only occur in the long term (e.g., osteoporosis, clostridium difficile or respiratory infections caused by proton pump inhibitors) without additional costs during hospitalization. These pharmaceutical interventions remain relevant and could be valuable to studies examining ROI calculations from a societal perspective. It is worth noting that DRP detection also aims to impact clinical and human factors. Although identifying a DRP may not only save money, it can also improve the patient’s quality of life.

Sensitivity analyses showed that our economic model was robust. The break-even point demonstrated that intervening solely on low-probability or low-cost ADEs was worth the effort financially.

Our study suggests that pharmacists need to prioritize their interventions on risky DRPs to avoid drug-related morbidity costs. They should focus their interventions primarily on patients who pose a high clinical and financial risk to the hospital, regardless of whether they are common or uncommon. (e.g., fragile populations, poly-morbidity/medication).

Pharmacists should also focus on drugs with a high risk of acute ADEs or medication errors (e.g., drugs with narrow therapeutic range). To achieve this, they should seek the support of digital tools to help identify these situations. New existing systems can flag patients at risk of ADEs by using different triggers in electronic patient records. Moreover, they are cost-effective [52–56].

Unlike other studies [8, 9, 21, 31, 39, 57], ours did not include direct costs saved in medication (e.g., an inpatient’s prescribed expensive compound to a less expensive one, switching from an intravenous to an oral form, or using a biosimilar instead of an original drug). If other types of costs, such as humanistic, indirect, and intangible costs, were considered in this analysis, the savings would have been even increased.

Despite our innovative cost calculation method, the present study had some limitations. The data sample for measuring costs associated with different categories of DRPs was limited. A larger sample size would have resulted in more accurate, realistic costs. Random data selection from such a small sample may not be sufficiently representative of the actual distribution of identified risk situations. The expert panel may also have influenced the costs used since other evaluators might have chosen differently at this stage of the project. The precise attribution of avoided costs to a DRP is highly variable because it depends on the DRP’s actual clinical impact, which is difficult to predict. For example, we calculated the savings from detecting a *drug interaction* DRP to be € 725. Other studies estimated these avoided costs to be between € 285 and € 14,943 [31, 33, 34, 38]. The way in which costs are quantified can greatly influence this value. With this model, each DRP was linked to a single potential ADE. Some clinical situations, might involve multiple ADEs stemming from one DRP, potentially increasing the avoided cost. This overlapping impact was not assessed in this analysis. The precise definition of pharmacists’ clinical interventions and categories of DRP may also influence cost calculations and could explain interstudy cost variations. There are no standardized definitions of these interventions. Finally, only the pharmacists' direct salary costs were used for cost investments calculations. Including indirect costs would lower the net benefit and ROI.

Conducting a comparative cost‒benefit study is recommended to assess the best financial impact of clinical pharmacists. It will enable an estimation of the actual differences in hospitalization costs between inpatients with DRPs undergoing or not undergoing medication review*.* Previous research teams have already conducted cost‒benefit analyses and cost-minimization studies to evaluate the cost-effectiveness of pharmacists' interventions [29, 52, 58–60]. Many cost-effectiveness publications have utilized cost-utility studies to measure benefits in quality-adjusted life years [35, 59, 61–68]. Alternatively, using prospective randomized controlled studies or before-and-after economic studies could enhance the measurement of avoided costs, as demonstrated by some research groups [57, 58, 67, 69–71]

## Conclusion

The present study showed the positive economic impact and favourable cost–benefit ratio of a medication review intervention performed by clinical pharmacists during internal medicine ward rounds. An innovative calculation model used real hospital costs to estimate the savings that a pharmacy department could deliver to a hospital with a pharmacist-led ADE prevention program—this could be a cost justification method in the pharmacy department’s budgeting.

It thus seems sensible for a hospital to invest in clinical pharmacists who can contribute to reducing health care costs by preventing ADEs. Standardized methods of demonstrating the economic impact of clinical pharmacists’ interventions and how to value an ADE are needed to make reliable use of increasing amounts of electronic hospital data.

To consolidate these findings, it would be interesting to carry out a complete economic cost–benefit study, evaluating the cost impacts of long-term ADEs or assessing the savings that can be made through other patient-centred pharmaceutical interventions.

### Supplementary Information

Below is the link to the electronic supplementary material.

Supplementary file1 (DOCX 30 kb)

Supplementary file2 (DOCX 20 kb)
