# Supplementary material for: Financial impact of medication reviews by clinical pharmacists to reduce in-hospital adverse drug events: a return-on-investment analysis
Source: Int J Clin Pharm. 2024 Feb 5;46(2):496–505. doi: 10.1007/s11096-023-01683-w (PMC10960916; doi:10.1007/s11096-023-01683-w)
Supplement: Supplementary file 1 — Supplementary file1 (DOCX 30 kb) [file 11096_2023_1683_MOESM1_ESM.docx]

**Supplementary Material n°1**

Table 1: Examples of drug related problems (DRPs) identified and solved by pharmacists to calculate avoided cost by DRP category.

|  | DRP  identified and solved | Recommended pharmaceutical intervention | Potentially avoided harm | Potential ADE | Probability of ADE occurrence | Institutional median cost  (€, 2017) |
| --- | --- | --- | --- | --- | --- | --- |
| Drug interaction | Discontinuation of posaconazole/clarithromycin in a immunosuppressed patient after a stem cell transplant: cessation of the PK interaction with ciclosporin interaction leading to a reduction in ciclosporin levels. | Make a ciclosporin levels 3 days after stopping clarithromycin and posaconazole and increase ciclosporin dose if necessary | Ciclosporin treatment failure | Recurrent GVHD | 0.1 | 27,942 |
| Drug interaction | PK drug interaction between amiodarone and dabigatran in a patient treated for atrial fibrillation: 50% increase in dabigatran AUC. | Switch for another DOAC with no interaction (apixaban) or OAC (acenocoumarol) | Dabigatran overdose | Intracerebral haemorrhage | 0.4 | 15,591 |
| Drug interaction | Ibuprofen-olmesartan-furosemide PD interaction: blockage of the two compensatory mechanisms in the nephron (afferent arteriolar dilatation and efferent arteriolar constriction) | Discontinuation of NSAIDs and switch to opioids to treat pain | Increased nephrotoxicity | Acute renal failure | 0.4 | 9,490 |
| Drug interaction | Paroxetine-domperidone PK interaction: increased risk of long QT  Additional risk factor is hypokalaemia (3.1mmol/L) | Change domperidone for metoclopramide or check ECG | Long QT | Long QT | 0.1 | 7,253 |
| Drug interaction | Cessation of smoking during hospitalization in a patient with epilepsy.  Discontinuation of tobacco-olanzapine PK interaction (CYP1A2 induction) leading to a gradual increase in olanzapine levels. | Monitor alertness and reduce olanzapine dose if necessary | Increased olanzapine side effects | Epileptic seizure | 0.1 | 5,609 |
| Inadequate dosage | Rivaroxaban underdosage:  Patient treated with 15 mg rivaroxaban once daily in the context of atrial fibrillation and renal function at 53 ml/min/1.73m^2^ (CKD-EPI formula) and 55ml/min (Cockcroft and Gault) | Increase the dose of rivaroxaban to 20 mg once daily | Heart embolism | Ischemic stroke | 0.01 | 10,886 |
| Inadequate dosage | Paracetamol overdose:  A dose of 4g per day for chronic lower back pain in a patient with chronic alcoholism. | Reduced paracetamol dose to a maximum of 2g per day. | Hepatotoxicity | Toxic liver disease with acute hepatitis | 0.1 | 5,657 |
| Inadequate dosage | Amoxicillin and clavulanate underdosing: Patient treated orally with 312.5 mg every 12 hours for a toe infection (initially adapted to renal function). Acute renal impairment resolved and creatinine clearance > 30 mL/min. | Increased amoxicillin and clavulanate from 312.5 mg to 625 mg three times a day after resolution of ARI. | Undertreated infection | Osteomyelitis | 0.1 | 16,151 |
| Inadequate dosage | Gentamicin underdosing:  Patient treated for an endocarditis with gentamicin 0.5 mg/kg every 8 hours with moderate renal impairment (Clcr 40 ml/min) (synergia with flucloxacillin)  Therapeutic Drug Monitoring showed:  high residual rate (1.5mg/L) (risk of toxicity); low peak rate (2.5 mg/L) (risk of inefficiency) | For a higher peak rate (target 5-7mg/L) and lower residual rate (target <1mg/L).  Change the frequency of administration and daily dose to 1 mg/kg every 24 hours | Endocarditis progression. Renal impairment. | Acute Renal Failure | 0.1 | 9,490 |
| Inadequate dosage | Metoclopramide overdose:  Patient with end-stage renal disease (eGFR <10 ml/min) receiving metoclopramide 10 mg three times a day for nausea and vomiting. | Reduce the dose by 75% to 5 mg three times a day | There is a significant risk of renal accumulation and metoclopramide toxicity (Extrapyramidal syndrome, Neuroleptic malignant syndrome, Confusion etc.) | Delirium | 0.4 | 9,346 |
| Improper drug selection | Diltiazem in a patient with acute heart failure. | Stop diltiazem and titrate a beta-blocker (metoprolol or bisoprolol) | Worsening of heart failure | Acute Heart Failure | 0.1 | 10,931 |
| Improper drug selection | Flunitrazepam in an 85-year-old patient | Change for benzodiazepine without active metabolites and eliminated essentially by phase 2 (lorazepam, oxazepam) | Cumulative sedative effect | Fall | 0.1 | 11,254 |
| Improper drug selection | 84-year-old patient who had a fall with CT and ACS and was on gliclazide 60mg bid, linagliptin 5mg q.d and metformin 500mg q.d: gliclazide and metformin were stopped because of an acute renal failure (Clcr 39). | Do not reintroduce gliclazide as there is a risk of hypoglycaemia and according to STOPP/START criteria it is contraindicated in elderly patients. Choose another antidiabetic drug (e.g.GLP1 analogue or SGLT2 inhibitor) | Hypoglycaemia | Hypoglycaemia | 0.2 | 3,299 |
| Improper drug selection | Patient with unstaged COPD with FEV_1_ 77% of predicted who is treated with Seretide® (fluticasone + salmeterol) (CSI + LABA): CSI questioned as patient not known to have had 2 outpatient exacerbations or 1 inpatient exacerbation within 1 year. In addition, it is introduced in patients with a FEV_1_ < 60% of predicted. If the patient is known to have pneumonia, then ICS is contraindicated because it increases the risk of pneumonia. | Prefer LAMA combined with LABA to prevent exacerbations and hospitalisations (GOLD guidelines) | Pneumonia | Pneumonia | 0.1 | 8,973 |
| Improper drug selection | Young patient admitted to hospital with pyelonephritis on morphine 10mg qid for headaches. | Switch to ibuprofen | Constipation | Constipation | 0.1 | 2,862 |
| Drug use without an indication | Aspirin without indication, in patient with thrombocytopenia and poorly controlled hypertension. | Stop acetylsalicylic acid | Combined risk of haemorrhage (thrombocytopenia and poorly controlled hypertension) | Intracerebral haemorrhage | 0.4 | 15,591 |
| Drug use without an indication | Pramipexole in a patient with no indication | Stop pramipexole | Dizziness and sedation | Orthostatic hypotension | 0.1 | 6,662 |
| Drug use without an indication | Esomeprazole without indication (acetylsalicylic acid stopped) in a patient with known osteoporosis and recent fracture. | Stop esomeprazole | Increased risk of fracture in patients with osteoporosis | Femoral neck fracture | 0.1 | 17,384 |
| Drug use without an indication | Patient on sodium polystyrene sulfonate for hyperkalaemia who is currently hypokalaemia | Stop sodium polystyrene sulfonate | Hypokalaemia | Hypokalaemia | 0.1 | 2,622 |
| Drug use without an indication | Acetylsalicylic acid (secondary prevention of a myocardial infarction) not indicated anymore (>12 months after acute event) in patient already treated with rivaroxaban for an atrial fibrillation. | Proposal to stop acetylsalicylic acid | Risk of bleeding | Upper digestive bleeding | 0.01 | 5,284 |
| Untreated indication | 77-year-old patient, known for ischemic heart disease, hypertension, and type 2 diabetes, hospitalized for septic shock of pulmonary origin. The patient is typically on clopidogrel, which has not been re-prescribed | Start clopidogrel | Myocardial infarction | Myocardial infarction | 0.1 | 12,604 |
| Untreated indication | Patient usually treated with clomipramine 75mg once daily at home who is missing at hospitalization | Start clomipramine | Increase in pain | Agitation | 0.1 | 10,264 |
| Untreated indication | Patient who has just suffered a ACS and beta-blocker (metoprolol) is missing. | Start metoprolol | Lack of cardioprotective effect | Angina pectoris | 0.1 | 5,647 |
| Untreated indication | Patient on acenocoumarol at home because of the mechanical valve.  At the time of hospital admission, the INR was at 11 and acenocoumarol was stopped: currently INR 1.83 (5 days without acenocoumarol or other anticoagulation treatment.)  Acenocoumarol needs to be reintroduced. | Start enoxaparin 1mg/kg/ bid plus acenocoumarol qd with INR monitoring. | Risk of cardiac embolism | Ischemic stroke | 0.4 | 10,886 |
| Untreated indication | Patient with hyperuricemia who was taking allopurinol 300mg once daily at home, not prescribed during hospitalization. Reintroduce allopurinol at a dose adjusted for renal function. | Start allopurinol | Hyperuricemia | Gout flare | 0.1 | 7,143 |

(ACS) Acute Coronary Syndrome, (AUC) Area Under the Curve, (bid) twice a day, (Clcr) Creatinine Clairance, (COPD)Chronic Obstructive Pulmonary Disease, (CSI) Corticosteroid Inhaler, (CT) Craniocerebral Trauma, (DOAC) Direct Oral Anticoagulation, (eGFR) estimated Glomerular Filtration Rate, (FEV) Forced Expiratory Volume, (GVHD) Graft-Versus-Host Disease , (HFrEF) Heart Failure with Reduced Ejection Fraction, INR (International Normalized Ratio), (LABA) Long-Acting Beta-Agonist, (LAMA) Long-Acting Muscarinic Antagonist, (OAC) Oral Anticoagulation, (PK) pharmacokinetic, (PD) pharmacodynamic, (qd) once a day, (tid) three times a day,
