# Supplementary material for: Financial impact of medication reviews by clinical pharmacists to reduce in-hospital adverse drug events: a return-on-investment analysis
Source: Int J Clin Pharm. 2024 Feb 5;46(2):496–505. doi: 10.1007/s11096-023-01683-w (PMC10960916; doi:10.1007/s11096-023-01683-w)
Supplement: Supplementary file 2 — Supplementary file2 (DOCX 20 kb) [file 11096_2023_1683_MOESM2_ESM.docx]

**Supplementary Material n°2**

Table 1: Adverse drug events identified by the expert panel, with their ICD-10 code, the number of hospital stays used for the median calculation, and their related institutional median cost

| ADE | ICD-10 | Hospital stays number  (2017) | Institutional median cost  (€, 2017) |
| --- | --- | --- | --- |
| Acute Heart Failure | I50 | 1548 | 10,931 |
| Acute Renal Failure | N17 | 144 | 9,490 |
| Agitation | R45.1 | 10 | 10,264 |
| Angina pectoris | I20 + I25 | 299 | 5,647 |
| Atrial fibrillation with rapid ventricular response | I48 | 411 | 4,642 |
| Bradycardia | R00.1 | 39 | 5,828 |
| Cholangitis | K83.0 | 32 | 9,393 |
| Clostridium colitis | A04.7 | 58 | 9,948 |
| Community acquired pneumonia | J13 + J14 + J15 + J16 + J18 | 1095 | 8,973 |
| Constipation | K59.0 | 36 | 2,862 |
| Deep vein thrombosis | I80.1 + I80.28 + I80.3 | 29 | 5,715 |
| Delirium | F05 | 214 | 9,346 |
| Epileptic seizure | G40 + G41 | 403 | 5,609 |
| Extrapyramidal syndrome | G25.8 + G25.9 | 15 | 11,298 |
| Fall | R26.9 | 352 | 11,254 |
| Femoral neck fracture | S72.0 | 410 | 17,384 |
| Gastroenteritis / colitis | K52.1 | 42 | 9,047 |
| Gout flare | M10 | 49 | 7,143 |
| Hematuria | R31 | 54 | 2,698 |
| Hyperglycaemia | R73.9 + E11.90 +E13.90 + E14.90 | 30 | 3,911 |
| Hyperkalaemia | E87.5 | 144 | 8,126 |
| Hypertensive crisis | I10.01 + I10.91 + I11.01 + I12.01 + I13.11s + I13.21 + I15.81 | 54 | 3,518 |
| Hypoglycaemia | E16 | 7 | 3,299 |
| Hypokalaemia | E87.6 | 26 | 2,622 |
| Intracerebral haemorrhage | I60, I61 & I62 | 289 | 15,591 |
| Ischemic stroke | I63.1 + I63.2 + I63.3 + I63.4 + I63.5 | 753 | 10,886 |
| Long QT syndrome | R94.3 | 14 | 7,253 |
| Lower digestive bleeding | K62.5 | 27 | 5,374 |
| Metrorrhagia | N92.0 + N92.1 | 9 | 3,627 |
| Myocardial infarction | I21 | 611 | 12,604 |
| Orthostatic hypotension | I95.2 | 10 | 6,662 |
| Osteomyelitis | M86 | 71 | 16,151 |
| Peptic ulcer disease | K25.4 + K25.5 + K25.6 + K25.7 + K26.4 + K26.5 + K26.6 + K26.7 | 35 | 12,768 |
| Pyelonephritis | N10 | 203 | 6,723 |
| Recurrent Graf-Versus-Host-Disease (GVHD) | T86.0 | 39 | 27,942 |
| Respiratory failure | J96.0 | 124 | 11,974 |
| Rhabdomyolysis | T79.6 + T46.6 | 22 | 11,033 |
| Serotonin syndrome | T43.2 | 3 | 591 |
| Somnolence | R40.0 | 61 | 798 |
| Toxic liver disease with acute hepatitis | K71.2 | 10 | 5,657 |
| Upper digestive bleeding | K92.2 | 56 | 5,284 |
| Variceal hemorrage | I85.0 | 6 | 9,307 |

*ADE, Adverse Drug Event; ICD, International Classification of Diseases;* €*, EUROS*
